# Evaluation of ductal carcinoma in situ grade via triple-modal molecular imaging of B7-H3 expression

**DOI:** 10.1038/s41523-020-0158-y

**Published:** 2020-04-29

**Authors:** Sunitha Bachawal, Gregory R. Bean, Gregor Krings, Katheryne E. Wilson

**Affiliations:** 1 Department of Radiology, Molecular Imaging Program at Stanford, Stanford University, School of Medicine, Stanford, CA USA; 2 Department of Pathology, Stanford University, School of Medicine, Stanford, CA USA; 3 Department of Pathology, University of California San Francisco, San Francisco, CA USA

**Keywords:** Cancer imaging, Breast cancer, Tumour biomarkers

## Abstract

Ductal carcinoma in situ (DCIS) will account for 62,930 cases of breast cancer in 2019. DCIS is a pre-invasive lesion which may not progress to invasive carcinoma, yet surgery remains the mainstay treatment. Molecular imaging of a specific marker for DCIS grade for detection and active surveillance are critically needed to reduce potential overtreatment. First, breast cancer marker B7-H3 (CD276) expression was evaluated by immunohistochemical staining in 123 human specimens including benign epithelium (H-score 10.0 ± 8.2) and low (20.8 ± 17.7), intermediate (87.1 ± 69.5), and high (159.1 ± 87.6) grade DCIS, showing a positive association with DCIS nuclear grade (*P* < 0.001, AUC 0.96). Next, a murine DCIS model was combined with ultrasound molecular imaging of B7-H3 targeted microbubbles to differentiate normal glands from those harboring DCIS (*n* = 100, FVB/N-Tg(MMTVPyMT)634Mul, AUC 0.89). Finally, photoacoustic and fluorescence molecular imaging with an anti-B7-H3 antibody-indocyanine green conjugate were utilized for DCIS detection (*n* = 53). Molecular imaging of B7-H3 expression may allow for active surveillance of DCIS.

## Introduction

Breast cancer remains the second leading cause of cancer-related death in women with ~268,600 new cases and 41,760 deaths predicted in 2019^1^. Widely implemented advanced screening methods, such as mammography, ultrasound, and magnetic resonance imaging, have significantly reduced breast cancer mortality due to earlier detection of carcinomas and, increasingly, pre-invasive lesions such as ductal carcinoma in situ (DCIS) and earlier precursors^2–4^. With an estimated 62,930 new cases in 2019, DCIS now accounts for approximately 20% of all breast cancers detected during screening exams^1^. Once diagnosed, the vast majority of patients with DCIS undergo treatment including breast-conserving surgery with radiation or mastectomy. However, it is estimated that only 16% of low grade DCIS and 60% of high-grade DCIS cases will progress to invasive carcinomas^2^. Given this wide range of biologic behavior, it is debated whether there is a role for “watchful waiting” for low-risk DCIS, and large clinical trials to determine the best treatment options are currently underway^5^. Currently, only a select minority of patients adopt a watchful waiting approach with regular imaging surveillance and possibly hormonal therapy. For the cases that will never progress to invasive disease, overtreatment of DCIS comes with its own morbidity risks^6^ and represents a large, and potentially unnecessary, burden on the US medical system^2,3,5^. Currently, over $800 million is spent annually on DCIS surgical and medical treatment^7^. Therefore, if a cost-effective and noninvasive method was available to accurately risk-stratify and monitor DCIS for signs of progression^7^, clinicians and patients might make more informed decisions on treatment, reducing patient morbidity and healthcare costs.

Ultrasound (US) molecular imaging, which utilizes molecularly targeted microbubbles (MBs), has recently been explored as a potential method to increase the specificity of ultrasound breast screening, particularly in women with dense breast tissue^8–10^. Microbubbles are small (1–2 µm), non-toxic, lipid shell encapsulated gas bubbles which have targeting moieties attached to the surface. These MBs can circulate within the vasculature^11,12^ and attach to molecular targets associated with neoangiogenesis, one of the hallmarks of cancer. MB binding can then be detected noninvasively with contrast-enhanced ultrasound imaging (CEUS), representing a cost-effective, real-time, and noninvasive screening method. Previously, the use of US molecular imaging has been shown to be able to detect invasive breast carcinomas both clinically^13^ and preclinically^14–17^. US molecular imaging represents an ideal method to monitor DCIS for progression, if an ideal molecular target can be determined.

Previously, B7-H3 (CD276), a member of the B7 family of immunoregulators^18,19^, was found to be specifically overexpressed in the four molecular subtypes of breast cancer (luminal A, luminal B, HER2-enriched and triple-negative), and insignificantly expressed in benign epithelium and various fibrocystic changes^15^. B7-H3 expression is correlated with worse disease outcomes and may play multiple roles in cancers beyond immune regulation, participating in progression, proliferation, and metastasis^20–23^. B7-H3 shows both epithelial and endothelial expression, making it an ideal target for various contrast agents that bind either in the vasculature or on the cancer cells^24^. The expression of B7-H3 in DCIS may not only allow for stratification between low and high-grade lesions, which have different progression rates, but monitoring expression levels over time may allow clinicians to intervene with progression as appropriate. Active surveillance may reduce unnecessary prophylactic procedures, patient morbidity, and burden to the healthcare system.

Treatment of early disease typically includes less aggressive treatment, such as breast-conserving surgery. For optimal surgical outcomes, it is imperative that the surgeon is able to visualize the borders of the primary tumor, including involvement of DCIS, to minimize recurrence with negative margins^25^. Currently, almost one-quarter of patients will undergo additional surgical interventions due to positive or insufficient surgical margins^25^. Therefore, a real-time, high resolution, and highly specific method for intraoperative tissue assessment is critically needed.

It was shown previously that combined photoacoustic (PA) and fluorescent molecular imaging using an anti-B7-H3 antibody-near infrared dye (indocyanine green) conjugate (B7-H3-ICG) could differentiate clinically actionable breast carcinomas from normal mammary tissues in a transgenic murine model of breast cancer^24,26^. Photoacoustic imaging, utilizing the photoacoustic effect of generating sound waves subsequent to absorption of nanosecond pulses of light^27,28^, allows for high-resolution imaging of optical absorption at depth (up to 5 cm), while fluorescence imaging, lacking background signal, provides high sensitivity to the contrast agent and mimics a surgeon’s field of view. In addition, as optical absorption is wavelength dependent, PA imaging allows for spectral analysis of relative concentrations of tissue chromophores in the tissue, suppressing background signal, and allowing for highly specific imaging of exogenous contrast agents^27,29,30^. Combined with a “smart” contrast agent, such as B7-H3-ICG, which undergoes endocytosis-mediated shifts in optical absorption spectra detectable by PA imaging^24^, these modalities may represent the optimal method to guide surgeons in intraoperative assessment of tumor margins, reduce the occurrence of positive margins and the need for additional surgical procedures, and improve patient outcomes.

**Fig. 1:**
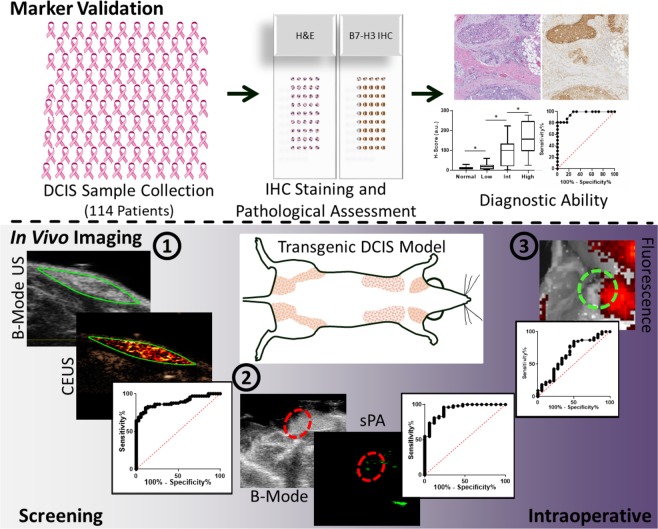
Study Overview. This study consisted of several parts. First, (top) samples of normal and low-, intermediate-, and high-grade DCIS were collected from 114 patients with up to 3 samples per patient. The patient samples were processed into a tissue microarray, underwent hematoxylin and eosin or B7-H3 immunohistochemical staining on adjacent slides, and were scored for B7-H3 intensity and percent staining. Histology scores were compared with pathology reports to surmise the diagnostic ability of the B7-H3 marker to differentiate low and high-grade DCIS. Next, (bottom) triple-modal molecular imaging of DCIS lesions in a transgenic mouse model was done in screening and intraoperative scenarios including ultrasound molecular imaging with B7-H3 targeted microbubbles and photoacoustic and fluorescence molecular imaging with an indocyanine green conjugated anti-B7-H3 antibody. Imaging signal was compared with histology to estimate detection ability of the imaging modalities.

The purpose of this study is multifold as depicted in Fig. 1. First, an extensive human tissue exploration of B7-H3 expression in normal mammary tissue, and low, intermediate, and high-grade DCIS lesions is undertaken. Next, ultrasound molecular imaging combined with B7-H3 targeted microbubbles is investigated for its ability to detect DCIS in a transgenic mouse model of breast cancer development for screening purposes. And finally, photoacoustic and fluorescence molecular imaging modalities, together with a B7-H3-specific antibody-near infrared dye conjugate, are assessed for detection of DCIS involvement at tumor margins in a preclinical, intraoperative scenario.

## Results

### Human B7-H3 expression in normal breast epithelium and DCIS

**Fig. 2:**
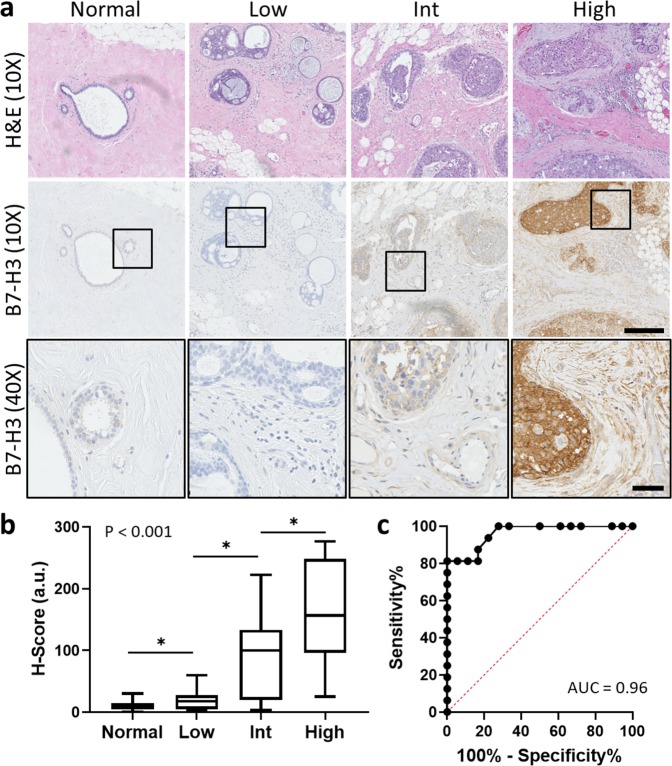
Analysis of B7-H3 expression with immunohistochemical (IHC) staining in normal human mammary tissue, and low, intermediate, and high-grade DCIS. **a** (Top) Representative 10X H&E micrographs of normal human breast tissue, and low, intermediate, and high-grade DCIS. (Middle) Corresponding sections with B7-H3 IHC staining showing increased expression with higher grade. Scale bar = 250 µm. (Bottom, insets) High magnification insets (×40) of B7-H3 IHC staining. Scale bar = 50 µm. **b** Box plot showing distribution of H-Scores (HS) from normal breast tissue (*n* = 57, HS = 10.0 ± 8.2), low grade DCIS (*n* = 18, HS = 20.8 ± 17.7), intermediate grade (*n* = 23, HS = 90.6 ± 68.7), and high-grade DCIS (*n* = 16, HS = 159.1 ± 87.6). All groups show statistical difference from each other (*P* < 0.001). Box plot follows Tukey rules. **c** Receiver operating curve depicting the ability of B7-H3 expression visualized by IHC to differentiate between low and high-grade human DCIS (AUC = 0.96, 95% CI 0.91, 1.00).

B7-H3 expression was assessed via immunohistochemical staining on human tissues including normal epithelium (*n* = 57) and three nuclear grades of DCIS: low (*n* = 18), intermediate (*n* = 23), and high (*n* = 16). B7-H3 expression was predominantly membranous. Overall, B7-H3 expression was significantly (*P* < 0.001) higher in high-grade DCIS (H-Score = 159.1 ± 87.6) compared with normal tissue (H-Score = 10 ± 8.2), low grade DCIS (H-Score = 20.8 ± 17.7), and intermediate grade DCIS (H-Score = 87.1 ± 69.5). Intermediate and low grades were found to have statistically significantly higher expression of B7-H3 than normal mammary tissues (*P* < 0.001). ROC analysis indicated that B7-H3 immunostaining could distinguish high grade from low grade DCIS with an AUC of 0.96 (95% CI 0.91, 1.00), and high grade from intermediate grade DCIS with an AUC of 0.73 (95% CI 0.57, 0.89) for all tissues sampled. Representative immunohistochemical staining images and summary graphs are depicted in Fig. 2.

### Histopathological analysis of murine mammary tissue

**Fig. 3:**
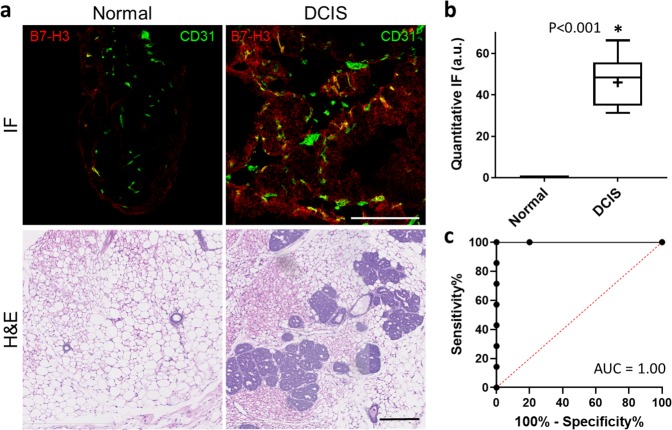
Quantitative immunofluorescence (QIF) analysis of murine B7-H3 expression in normal mammary tissue and glands containing DCIS. **a** Representative immunofluorescence (20×, top) and H&E (10×, bottom) stained normal murine mammary tissues (left) and those containing DCIS (right). Scale bars = 200 μm. **b** Box plot showing quantitative IF composite scores (a.u.) of normal breast tissue (*n* = 5, CS = 0.01 ± 0.02 a.u.) and DCIS (*n* = 7, CS = 45.96 ± 12.76 a.u.). Box plot follows Tukey rules. DCIS tissues have statistically significantly higher B7-H3 staining than normal murine tissue both on the endothelial and epithelial cells (*P* < 0.001). **c** Receiver operating curve depicting the ability of murine B7-H3 expression visualized by QIF to differentiate between normal and DCIS containing tissues (AUC = 1.00, 95% CI 1.00, 1.00).

B7-H3 expression in endothelial cells associated with normal murine mammary tissue and those demonstrating DCIS was assessed by quantitative immunofluorescence staining. Normal mammary glands showed minimal endothelial expression of B7-H3 as measured by histology composite score (CS = 0.01 ± 0.02 a.u.) while DCIS containing tissues had statistically significantly higher endothelial expression (CS = 45.96 ± 12.76 a.u., *P* < 0.001). Similarly, ROC analysis showed an AUC of 1.00 (95% CI 1.00, 1.00) for the ability to distinguish disease state by QIF staining. Representative micrographs and summary graphs are presented in Fig. 3.

### Ultrasound molecular imaging of murine DCIS

**Fig. 4:**
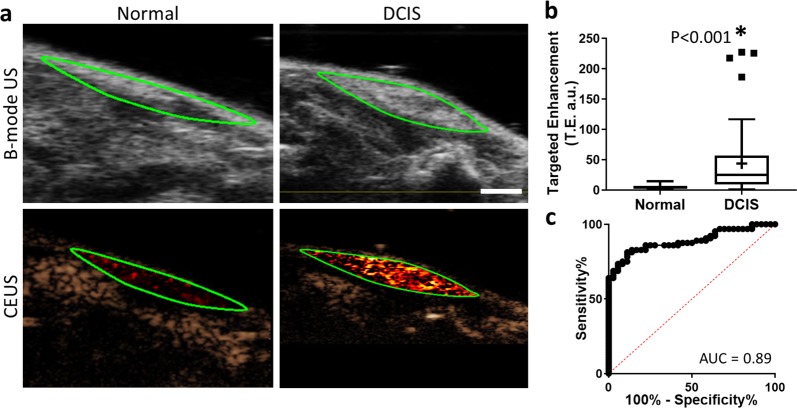
Contrast-enhanced molecular ultrasound (CEUS) imaging of B7-H3 expression in normal mammary glands and DCIS with B7-H3 targeted microbubbles (MB_B7-H3_). **a** Representative B-mode (top) and CEUS (bottom) images of murine mammary glands with either normal (left) or DCIS (right) tissues (green outline) enhanced with MB_B7-H3_. Scale bar = 2 mm. **b** Box plot of targeted enhancement (T.E., a.u.) of normal mammary glands (*n* = 36, TE = 5.0 ± 3.5 a.u.) and DCIS (*n* = 64, TE = 44.2 ± 53.3 a.u.). Box plot follows Tukey rules. Glands containing DCIS show statistically significantly higher CEUS signal with B7-H3 targeted microbubbles (*P* < 0.001). **c** Receiver operating curve depicting the ability of B7-H3 expression and CEUS with MB_B7-H3_ to differentiate between normal murine mammary glands and glands containing DCIS (AUC = 0.89, 95% CI 0.82, 0.95).

Next, the ability of CEUS molecular imaging to detect and differentiate B7-H3 expression in DCIS as compared with normal murine mammary tissues with the use of a B7-H3 targeted microbubble was assessed. After injection of MB_B7-H3_, glands with histologically confirmed DCIS (44.2 ± 53.3 a.u.) showed a statistically significant increase in CEUS signal (*P* < 0.001) compared with murine mammary glands containing normal tissues (5.0 ± 3.5 a.u.). ROC analysis of the ability of ultrasound molecular imaging to differentiate between normal tissues and DCIS provided an AUC of 0.89 (95% CI 0.82, 0.95). Sample B-mode and contrast-enhanced US molecular imaging images and summary graphs are provided in Fig. 4.

### Photoacoustic molecular imaging of murine DCIS

**Fig. 5:**
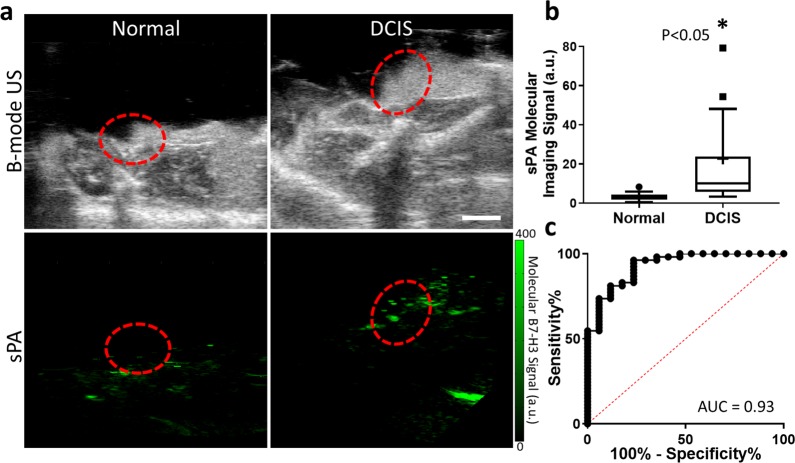
Spectroscopic photoacoustic (sPA) molecular imaging of a B7-H3 antibody-ICG conjugate (B7-H3-ICG) in normal mammary glands and glands containing DCIS. **a** Representative B-mode ultrasound (top) and sPA (bottom) images of murine mammary glands with either normal (left) or DCIS (right) tissues (red dashed circle) after injection with B7-H3 Ab-ICG. Scale bar = 2 mm. **b** Box plot of sPA molecular imaging signal (a.u.) distribution in normal mammary glands (*n* = 17, sPA = 3.2 ± 2.0 a.u.) and DCIS (*n* = 53, sPA = 22.7 ± 40.2 a.u.). Box plot follows Tukey rules. Glands containing DCIS show statistically significantly higher sPA molecular imaging signal (*P* < 0.05). **c** Receiver operating curve depicting the ability of B7-H3 expression imaged by sPA and B7-H3 Ab-ICG to differentiate between normal glands and glands containing DCIS (AUC = 0.93, 95% CI 0.86, 0.99).

Photoacoustic molecular imaging combined with an antibody-dye contrast agent was evaluated for its ability to image B7-H3 expression in DCIS and differentiate expression levels from normal mammary tissues in a transgenic model of breast cancer development. After injection of B7-H3-ICG, glands with histologically confirmed DCIS showed a statistically significantly higher average sPA molecular B7-H3 signal (22.7 ± 40.2 a.u.; *P* < 0.05) compared with normal murine mammary glands which showed an average of 3.2 ± 2.0 a.u molecular imaging signal. ROC analysis of the ability of PA molecular imaging to differentiate between normal tissues and those harboring DCIS provided an AUC of 0.93 (95% CI 0.86, 0.99). Representative images and summary graphs are presented in Fig. 5.

### Fluorescence molecular imaging of murine DCIS

**Fig. 6:**
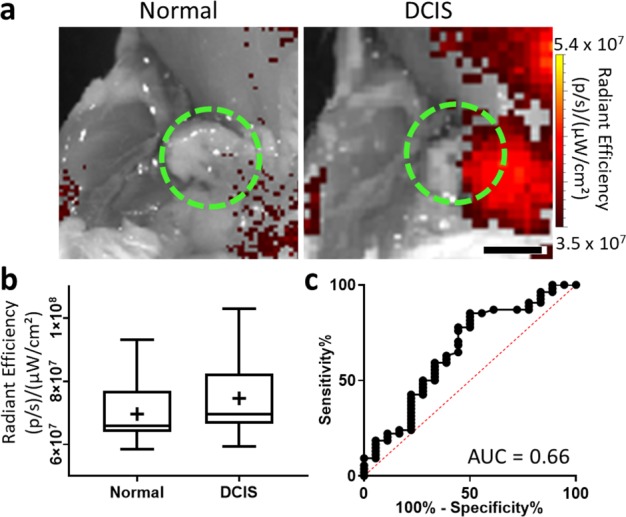
Fluorescence molecular imaging of a B7-H3 antibody-ICG conjugate (B7-H3-ICG) in normal murine mammary glands and glands containing DCIS. **a** Representative fluorescence images of murine mammary glands with either normal (left) or DCIS (right) tissues (green dashed circles) after injection with (B7-H3-ICG). Scale bar = 3 mm. **b** Box plot of fluorescence imaging signal (radiant efficiency (RE), (p/s)/(µW/cm^2^)) distribution of normal mammary glands (*n* = 18, RE = 7.0 × 10^7^ ± 1.0 × 10^7^ (p/s)/(µW/cm^2^)) and DCIS (*n* = 54, RE = 7.5 × 10^7^ ± 1.1 × 10^7^ (p/s)/(µW/cm^2^)). Box plot follows Tukey rules. Glands containing DCIS do not show statistically significantly higher sPA signal (*P* = 0.09). **c** Receiver operating curve depicting the ability of B7-H3 expression and fluorescence molecular imaging with B7-H3 Ab-ICG to differentiate between normal glands and glands harboring DCIS (AUC = 0.66, 95% CI 0.51, 0.81).

Next, fluorescence molecular imaging combined with the same antibody-dye contrast agent was evaluated for its ability to image B7-H3 expression in DCIS compared with normal mammary tissues. After injection of B7-H3-ICG, glands with histologically confirmed DCIS showed an increase in average of fluorescence signal (7.5 × 10^7^ ± 1.1 × 10^7^ (p/s)/(µW/cm^2^)) compared with normal mammary glands (7.0 × 10^7^ ± 1.0 × 10^7^ (p/s)/(µW/cm^2^)) but these differences were not statistically significant (*P* = 0.09). ROC analysis of the ability of fluorescence molecular imaging to differentiate between normal tissues and DCIS provided an AUC of 0.66 (95% CI 0.51, 0.81). Example fluorescence images and summary graphs are presented in Fig. 6.

## Discussion

Ductal carcinoma in situ, a pre-invasive lesion, now represents one-fifth of breast cancers detected in the United States. While only a portion of these cases will progress to invasive breast carcinomas, the clinical recommendation remains either breast-conserving surgery with radiation or mastectomy, potentially representing overtreatment in many cases. There are currently no clinically used methods or markers to differentiate DCIS as more or less likely to progress, let alone allow for noninvasive monitoring of lesions for signs of progression. In addition, DCIS involvement at the surgical excision margin is considered positive, and patients with positive margins undergo re-excision. Therefore, a cell surface marker that is not only highly specific for DCIS but can also correlate with nuclear grade and potential risk of invasion, as well as molecular imaging strategies to noninvasively and longitudinally detect and monitor these lesions, are critically needed.

Overall, this study consisted of three components. First, human clinical breast specimens including normal epithelium and DCIS of low, intermediate, and high grades were tested for B7-H3 immunohistochemical expression. Next, US molecular imaging (USMI) combined with a B7-H3 targeted microbubble was utilized in a screening capacity to image and differentiate DCIS lesions from normal mammary glands in a transgenic mouse model of breast cancer development. Finally, photoacoustic and fluorescence molecular imaging combined with a B7-H3-ICG antibody-dye contrast agent were utilized in an intraoperative scenario to detect small foci of DCIS in mammary glands of the murine breast cancer model.

Previously, B7-H3 has been shown by us and others to be expressed in numerous cancer types including breast^15,24,31,32^, and is generally considered to be correlated with poorer outcomes^20,33–35^. B7-H3 is a T-cell modulator, which in part functions to prevent immune invasion within the tumor stroma^21,31,36^. However, B7-H3 expression in early *in situ* breast carcinomas had not been characterized. Moreover, any association with grade, and therefore associated risk of progression to invasive disease, was unknown. It was found that low-grade lesions had statistically significantly lower B7-H3 protein expression by H-score (taking into consideration both intensity and percent tumor staining), than high-grade DCIS. In addition, low-grade lesions had moderately higher expression than normal epithelium. B7-H3 expression was found to be an excellent method to differentiate between low- and high-grade lesions with an AUC of 0.96. Membranous B7-H3 expression in tumor cells makes it an ideal target for various contrast agents that bind both intravascularly and on the cell surface. Therefore, B7-H3 could be an optimal marker to detect intermediate and high nuclear grade DCIS and serially monitor all grades of DCIS lesions for increasing B7-H3 expression using multiple noninvasive molecular imaging techniques.

A highly specific and sensitive, noninvasive imaging technique is critical to allow for monitoring of patients diagnosed with DCIS over time. Such a technique should be non-radiative, cost-effective, and rapid, all qualities of ultrasound imaging. Currently, ultrasound imaging is being studied for its ability to detect cancers in situ, and while B-mode ultrasound is highly sensitive, it lacks specificity for malignant lesions^8,10^. When ultrasound imaging is combined with molecularly targeted microbubbles there is a dramatic increase in the specificity of the modality. Here, USMI combined with an anti-B7-H3 microbubble was able to differentiate murine DCIS from normal mammary glands with an AUC of 0.89. Initial clinical trials using anti-VEGFR2 targeted microbubbles combined with USMI have been shown safe and hold great promise in cancer detection^13^. However, the VEGFR2 receptor has only shown a moderate ability to distinguish benign and malignant lesions (AUC of 0.71) in human tissues^37^ and only preliminary studies into the expression of VEGFR2 on high-grade DCIS have occurred^37,38^. Therefore, the USMI of the B7-H3 receptor and its high specificity and ability to differentiate between normal, DCIS, and invasive lesions represents an optimal modality for longitudinal monitoring of DCIS to help plan surgical treatment.

Aside from detecting and monitoring DCIS for screening purposes, it is also critical to be able to do so in the intraoperative setting to ensure negative margins. Significant research effort is currently dedicated to implementing fluorescence and photoacoustic imaging for intraoperative guidance^26,39–41^. Here, photoacoustic molecular imaging combined with the B7-H3-ICG contrast agent was shown to be able to detect small foci of DCIS in a murine model of breast cancer development. Direct correlation between imaging signal and histological stage (normal vs. DCIS) was determined, and photoacoustic molecular imaging was able to differentiate B7-H3-ICG accumulation within small (<1 mm) foci of DCIS from normal murine mammary glands with an AUC of 0.93. The high specificity of photoacoustic imaging of the B7-H3 agent arises from the dynamic optical absorption spectrum of the B7-H3-ICG when binding to its molecular target. Photoacoustic imaging has the sensitivity to detect the changes in the optical absorption spectrum and suppress background signal from blood and unbound agent and this ability was verified through a multi-control study^24,26^. While the required imaging depth was limited in this study due to the superficial and small nature of murine mammary glands, photoacoustic imaging was able to provide high-resolution images of optical contrast at depth within the glands. Currently, clinical photoacoustic systems are emerging on the market that are optimized for human application and imaging depths up to 5 cm^42–46^, making clinical photoacoustic imaging, especially during intraoperative scenarios, a feasible molecular imaging technique to detect and monitor DCIS in humans.

While photoacoustic imaging provides high-resolution images at depth within a surgical field, it remains a focal imaging scan that transects the imaging plane visible to the surgeon. Fluorescence imaging provides a wide field of view that corresponds directly to the surgeon’s, making the two modalities highly complementary for intraoperative molecular imaging. However, in this study fluorescence imaging of the B7-H3-ICG agent was not able to reliably detect significant differences between DCIS and normal tissues, indicating an early stage disease sensitivity limit for the modality in this application. Previously, the modality has been able to differentiate invasive carcinoma in a similar situation^26^. Fluorescence imaging has larger filter bandwidth (~40 nm) for excitation and emission, and therefore taking advantage of the shifting ICG spectrum is difficult for the modality compared with photoacoustic imaging, and consequently separating blood pool signal from B7-H3-specific signal proved difficult. Currently, fluorescence guided surgery is a large area of ongoing research with multiple applications and available agents being developed^39–41^. The familiar “bird’s eye view” of fluorescence imaging makes it highly compatible with surgical applications and user friendly for surgeons. The high sensitivity of fluorescence imaging can detect small amounts of tracer due to limited background signal, and fluorescence can easily detect areas of agent accumulation away from the primary site of interest, critical in surgical settings, a limitation of ultrasound and photoacoustic techniques. Fundamentally, when used together, photoacoustics and fluorescence may offer a high-resolution and high-sensitivity molecular imaging at depth and with a wide field of view all with one molecularly targeted contrast agent for multiple purposes including intraoperative guidance.

There are several limitations of this study. First, as most patients undergo treatment after diagnosis with DCIS, determining progression of disease or changes in B7-H3 expression with progression was not possible within the patient population studied here. Second, the murine model of breast cancer progression used in these studies is transgenic with a virus promoter of Polyoma Virus middle T antigen (PyVT). Therefore, mammary glands will always progress to invasive carcinomas with time and it is not possible to grade murine DCIS as in human disease. There is currently no natural model of murine DCIS to use to correlate B7-H3 expression levels with progression probabilities. Furthermore, disease development in this model is multifocal, which, even in early disease such as DCIS, could indicate higher disease burden within the mammary glands. Increased areas of DCIS could overestimate the ability of the imaging modalities to detect early disease. However, for the imaging studies here, each gland was analyzed as a single source of imaging signal, including the surrounding normal tissues, potentially minimizing molecular imaging signal from DCIS. Finally, there is no need to accommodate depth during in vivo mouse imaging studies due to their small size. Typically, optical modalities suffer from depth limitations, and while photoacoustics can image up to 5 cm deep, intraoperative use, as suggested here, highlights an optimal application due to reduced depth requirement.

In conclusion, the results of this study suggest that B7-H3 protein expression levels in human DCIS strongly correlate with nuclear grade and, therefore, likelihood of progression to invasive carcinoma. In addition, ultrasound, photoacoustic, and fluorescence molecular imaging techniques combined with appropriate B7-H3 targeted contrast agents were able to detect DCIS in a murine model of breast cancer development. In the future, these imaging strategies may allow for active surveillance of DCIS or detection of DCIS in an intraoperative scenario minimizing unnecessary surgical interventions.

## Methods

### Tissue microarray construction of human DCIS specimens

Human breast tissue samples were obtained retrospectively and were selected under an HIPAA compliant, Institutional Review Board-approved protocol at the University of California San Francisco which superseded the need for informed consent. A total of 57 samples of DCIS of low, intermediate, or high nuclear grade and an additional 57 cases of normal breast epithelium were evaluated utilizing tissue microarray (TMA). In brief, TMA cases were constructed from patient excisions after characterization by a dedicated breast pathologist. Three 2-mm punch biopsy tissue cores were obtained from each case for analysis. Positive and negative on-slide controls consisted of normal breast and invasive ductal carcinoma of no special type.

### Immunohistochemical staining of human DCIS specimens

Immunohistochemical staining and scoring of collected human samples was performed using anti-human B7-H3 antibody (AF1027, 1:300 dilution; R&D systems, MN) on 5 µm sections of paraffin-embedded breast tissues using heat induced antigen retrieval program at pH 9.0. A dedicated breast pathologist scored all immunohistochemically stained sections by H-score^47^, incorporating intensity and percent positive membranous staining (1 * % with low intensity + 2 * % with moderate intensity + 3 * % with strong intensity), resulting in ranges of values between 0 and 300. Replicate samples (1–3 per patient) were averaged. The H-scores from within each category (normal, low grade, intermediate grade, and high-grade DCIS) are presented as means ± standard deviation.

### Mouse model of DCIS

All experiments involving animals were approved by the Institutional Administrative Panel on Laboratory Animal Care at Stanford University. A transgenic mouse model of breast cancer (FVB/N-Tg(MMTVPyMT)634Mul) was used^48^. The MMTV-PyMT model closely recapitulates human disease by spontaneously progressing through several stages of breast cancer disease including hyperplasia, DCIS, and finally invasive carcinoma between 5 and 12 weeks of age, independently in each mammary gland. By 5–7 weeks most of the 10 mammary glands in a given animal will contain multifocal DCIS^14,48^. Glands that were found to contain either hyperplasia or invasive carcinoma, as assessed by ultrasound imaging and hematoxylin and eosin (H&E) staining, were excluded from the study. Normal mammary glands from littermates that were negative for the transgene were used as controls. Fur covering the mammary glands was removed with the use of depilatory cream. During US molecular imaging studies, mice were anesthetized with 2% isoflurane in oxygen flowing at 2 L/min. Heart rate, ECG, and respiration rate were monitored using an Advanced Physiological Monitoring Unit, and the body temperature was maintained at 37 °C with the use of a heated stages and pre-warmed ultrasound gel. For photoacoustic and fluorescence molecular imaging, animals were humanely euthanized immediately before imaging.

### Histopathological analysis of murine mammary tissue

Immediately after imaging, mammary tissues from transgene positive animals, and select mammary glands from transgene negative animals were excised, formalin-fixed and paraffin-embedded. Tissue sections (10 μm) were stained with H&E via standard protocol. Histological diagnosis of DCIS versus normal was rendered blinded to the imaging findings and age of the animal. Normal mammary tissue was comprised of primarily adipose inclusive of a few, well-organized ducts. DCIS was defined as an increase of acinar cell clusters with completely expanded intraductal epithelial proliferation^49^ and solid, cribriform and/or comedo architecture with cells demonstrating intermediate to high nuclear grade. Comedonecrosis was focally identified. Glands containing invasive carcinoma or hyperplasia (characterized by a relatively preserved ductal system with increased volume of glandular and acinar tissue^49^) were excluded from the study. Pathological assessment was used to classify mammary glands for molecular imaging analysis.

### Immunohistochemical staining and pathological scoring of murine DCIS

Murine B7-H3 expression of the transgenic animal model was confirmed with ex vivo immunofluorescence staining as described previously^15,24^. Briefly, mammary glands known to harbor DCIS (*n* = 7) and normal tissues (*n* = 5) on H&E-stained sections were split before fixation, frozen in optimal cutting temperature (OCT) compound, sectioned (10 μm), rinsed with PBS for 5 min, fixed with 4% paraformaldehyde for 10 min, and permeabilized with 0.05% Triton in PBS for 15 min. Sections were blocked with 5% BSA, 5% goat serum in PBS for 1 h at room temperature. Primary antibodies were applied (rabbit anti-mouse B7-H3 and rat anti-mouse CD31 antibodies (Abcam Inc.)) overnight at 4 °C at dilutions of 1:50 and 1:100, respectively, and were visualized with labeled secondary antibodies (AlexaFluor-488 conjugated goat anti-rabbit or AlexaFluor-546 anti-rat secondary antibodies (1:300) respectively) (Invitrogen, Grand Island, NY). Fluorescent images were acquired with a LSM 510 Meta confocal microscope (Zen 2009, Carl Zeiss) at ×200 magnification.

Though B7-H3 is expressed on both endothelial and epithelial cells in diseased murine mammary tissue^15,24^, only endothelial expression was analyzed to provide comparable ROIs between normal (no epithelial expression) and DCIS (epithelial expression). In random fields of view, vessels were visualized with CD31 staining and quantified using ImageJ (version 1.46r) software. ROIs were determined by converting to 8 bit images then applying a threshold to create binary images. These binary images were used as masks on the B7-H3 channel images, and fluorescence intensity was measured using mean fluorescence intensity (a.u.)^15^. Data are presented as means ± standard deviation and differences were assessed by a two-sided *t*-test, with *P* values of <0.05 considered indicative of statistically significant differences between the means. The ability of B7-H3 expression to differentiate normal glands and those glands demonstrating DCIS was examined with receiver operating characteristic (ROC) analysis presented as area under the curve (AUC) and 95% confidence intervals.

### Targeted microbubble preparation and ultrasound molecular imaging of murine DCIS

For ultrasound molecular imaging of B7-H3 expression in murine DCIS versus normal, animals with normal mammary glands (*n* = 4 mice, 36 glands shown to be normal on histology) or those suspected of containing DCIS due to age (*n* = 7, 64 glands with confirmed DCIS by histology) underwent molecular imaging immediately following injection of B7-H3 targeted microbubbles.

Commercially available preclinical streptavidin-coated, perfluorocarbon containing lipid-shelled microbubbles (VisualSonics, Toronto, Canada) were used to generate B7-H3-targeted microbubbles (MB_B7-H3_) as described previously^14,15^. Briefly, each vial of lyophilized streptavidin-coated microbubbles was suspended in 1 mL of sterile saline (0.9% sodium chloride). To add the targeting antibody to the surface, 6 μg of biotinylated rat anti-mouse B7-H3 antibodies ([M3.2D7] eBiosciences, San Diego, CA) were incubated with 5 × 10^7^ MBs for 10 min at room temperature. Bubbles were used immediately.

Targeted CEUS was performed in contrast mode using a dedicated small-animal high-resolution ultrasound imaging system (Vevo 2100; VisualSonics, Toronto, Canada) equipped with an 18 MHz transducer (MS250: lateral and axial resolution of 165 and 75 µm, 8 mm focal length, 10% transmit power, 0.2 mechanical index, 40 dB dynamic range). Mammary glands were imaged in the transverse cross section at the location of maximal gland diameter. To differentiate between the acoustic signal from adherent microbubbles and the signal from freely circulating microbubbles, destruction and replenishment curves were obtained as previously described^14,50^. Briefly, 4 min after injection of MB_B7-H3_, during which bubble binding has reached a steady state in the field of view, 200 frames of CEUS were captured. Next a destructive pulse (3.7 MPa, transmit power, 100%; mechanical index, 0.63) was delivered to clear all MBs within the field of view. Finally, the MB replenishment curve (indicative of freely circulating MBs) was monitored by collecting 200 additional frames of CEUS. Multiple glands can be imaged with a single injection of MBs ^14,15,51,52^.

### Preparation of indocyanine green labeled anti-B7-H3 antibody contrast agent

B7-H3-targeted antibody-dye conjugation and characterization was carried out as previously described^24,26^. Briefly, per batch, 100 µg B7-H3-targeted antibody [Abcam Inc., [EPNCIR122] (ab134161) in 200 µl PBS was incubated with a 20x molar equivalent of ICG-NHS (Intrace Medical, Co.) in 50 μl dimethyl sulfoxide (DMSO) to form bonds via NHS ester labeling of amino groups of the lysine peptides on the antibodies. Labeled antibodies were separated from free ICG dye using a Sephadex™ PD-10 gel filtration column as a visible fraction. Protein concentrations were determined through spectrophotometric analysis at 280 and 560 nm with correction factor^24,53^. Ab-dye conjugates were concentrated to 0.33 mg/ml in PBS using 30,000 Da molecular weight cutoff filters centrifuged at 2500 × *g* for 5 min. For photoacoustic and fluorescence molecular imaging techniques, each animal was injected via tail vein with 100 µl (33 µg) of the B7-H3-ICG contrast agent 24 h before imaging to allow for sufficient accumulation at the target sites^24,26^.

### Photoacoustic molecular imaging of murine DCIS

For photoacoustic molecular imaging of B7-H3 expression, animals with normal mammary glands (*n* = 2 mice, 17 glands) or those harboring DCIS (*n* = 6 mice, 53 glands histologically validated as DCIS) underwent molecular imaging before and 24 h after injection of the B7-H3-ICG contrast agent and immediately after humane euthanasia and removal of the skin over the mammary glands to allow for simulation of intraoperative imaging. The VisualSonics VevoLAZR (21 MHz transducer, lateral and axial resolution of 165 and 74 µm, respectively, 10 mJ/cm^2^ average fluence, 10 ns pulse width, 20 Hz pulse repetition frequency) was used to obtain transverse, single-plane ultrasound images to identify the maximal gland diameter and maintain imaging consistency between time points. Co-registered, multiwavelength (680–900 nm, 10 nm increments) photoacoustic images were then obtained. The transducer surface was maintained at 9 mm from the surface of the skin coupled with clear, bubble free, colorless gel to maintain even light illumination throughout the gland and imaging consistency. Images were saved in the .RAW format for subsequent processing.

### Fluorescence molecular imaging of murine DCIS

For fluorescence molecular imaging of B7-H3 expression, animals with normal mammary glands (*n* = 2 mice, 18 glands) or those harboring DCIS (*n* = 6, 54 glands histologically validated as DCIS) underwent molecular imaging 24 h after injection of the B7-H3-ICG contrast agent and immediately after humane euthanasia and removal of the skin covering the mammary glands to simulate intraoperative conditions. Fluorescence images were collected using the IVIS Spectrum Preclinical Imaging System (PerkinsElmer) in epifluorescence mode equipped with 710/30 nm and 820/20 nm filters for excitation and emission, respectively. Data were saved for subsequent image analysis.

### Analysis of in vivo molecular imaging data

All imaging data was evaluated after acquisition in random order by a researcher blinded to the histological classification of the mammary glands.

Ultrasound molecular imaging signal, corresponding to the volume of MB_B7-H3_ attached to B7-H3 receptors on the vasculature in the mammary gland, was measured using commercially available software (Vevo LAB, Version 1.7.2.7310, VisualSonics). ROIs were drawn over imaged mammary glands and the value of the differential targeted enhancement (T.E. (a.u.)) was calculated, defined as the average pre-destruction imaging signal (a.u.) divided by the average post-destruction signal (a.u.) as described previously^14,17,54^. Data are presented as means ± standard deviation.

Photoacoustic molecular imaging signal corresponding to relative B7-H3 expression levels was determined using a custom MATLAB-based linear least squares error classification algorithm that has been described in detail elsewhere^29,55,56^. Briefly, the co-registered B-mode ultrasound images were used to guide selection of a region of interest (ROI) over the whole mammary gland. Then, the relative concentrations of each photoabsorber (ICG, oxygenated, and deoxygenated hemoglobin) were calculated by comparing wavelength dependent PA signal changes to known optical absorption properties of the chromophores with linear regression techniques. Photoacoustic molecular imaging signal is displayed as the ratio of ICG signal 24 h after injection compared with before injection imaging within the ROI. Data are presented as means ± standard deviation.

Fluorescence molecular imaging signal, quantitatively measured and corresponding to B7-H3-ICG accumulation in the mammary gland, was evaluated using Living Image Software 4.0 (PerkinElmer). Fixed sized ROIs were placed over each mammary gland guided by co-registered photographs and average radiant efficiency (p/s//cm^2^/sr/µW/cm^2^) was measured. The background blood pool signal was measured with an ROI over the sternum. Fluorescence signal ratios were calculated as the radiant efficiency in the mammary gland over the background signal. Data are presented as means ± standard deviation.

Receiver operating curves (ROC analysis) were used to elucidate the ability of the various molecular imaging modalities and agents to differentiate normal tissues from DCIS and are presented as the AUC followed by the 95% confidence interval. Statistical significance was determine through two-sided *t*-tests with a *P* value <0.05 considered significant. All statistical analysis was performed using Prism GraphPad (Version 8.1.2) software. Associated manuscript data in Prism GraphPad file format are available from the metadata entry^57^.

## Supplementary information

Reporting-Summary

## Data Availability

The data generated and analyzed during this study are described in the following metadata record: 10.6084/m9.figshare.11968758^57^. Human histology images are not publicly available in order to protect patient privacy. All other imaging source data can be made available on reasonable request to the corresponding author. Human B7-H3 DCIS histology scoring data and all murine B7-H3 imaging summary data are available as part of the metadata record^57^.

## Code availability

The MATLAB-based data analysis scripts used to process the spectroscopic photoacoustic molecular imaging signal are available from the corresponding author upon reasonable request. Additional information can be found in reference^29^. All other data analysis used commercially available software described in Methods.
